# Trials and Treatments: An Update on Pharmacotherapy for Idiopathic Pulmonary Fibrosis

**DOI:** 10.3390/life13020486

**Published:** 2023-02-10

**Authors:** Lorraine Thong, Enda James McElduff, Michael Thomas Henry

**Affiliations:** 1 Department of Clinical Medicine, Trinity College Dublin, D08 W9RT Dublin, Ireland; 2 Department of Clinical Medicine, Royal College of Surgeons Ireland, D02 YN77 Dublin, Ireland; 3 Department of Respiratory Medicine, Cork University Hospital, T12 YE02 Cork, Ireland

**Keywords:** idiopathic pulmonary fibrosis (IPF), treatment, pharmacotherapy, anti-fibrotic, novel, adjunct, pathophysiology, etiology

## Abstract

Idiopathic pulmonary fibrosis (IPF) is a chronic and progressive fibrosing interstitial lung disease that occurs predominantly in the older population. There is increasing incidence and prevalence in IPF globally. The emergence of anti-fibrotic therapies in the last decade have improved patient survival though a cure is yet to be developed. In this review article, we aim to summarize the existing and novel pharmacotherapies for the treatment of IPF (excluding treatments for acute exacerbations), focusing on the current knowledge on the pathophysiology of the disease, mechanism of action of the drugs, and clinical trials.

## 1. Introduction

Idiopathic pulmonary fibrosis (IPF) is a chronic and progressive fibrosing interstitial lung disease that occurs predominantly in the older population. It is the most common cause of idiopathic interstitial pneumonias and was previously thought to be a rare disease, however, there is an increasing trend in incidence and prevalence rate globally [1]. Currently, the global incidence rate of IPF ranges from 0.2 to 93.7 per 100,000 per year, with higher incidences across Europe and America and lower incidences in Asia and South America [1]. Primary clinical manifestations of IPF include progressively worsening dyspnea and decline in lung function [2]. The diagnosis of IPF carries a high mortality rate and disease burden as severe as several cancers such as esophagus, pancreas, and prostate but with incongruent public awareness, screening, and management [3]. Prior to 2010, there was no increase in the survival rate of IPF patients, however, with recent therapeutic advances, there may have been a slight increase in the trend since [3]. 

Despite the current improved understanding and therapeutic advances in IPF, apart from lung transplantation, there are no other curative treatments for IPF. In this paper, we aim to summarize the current and novel pharmacotherapies available for the treatment of IPF. 

### 1.1. Pathophysiology and Etiology

The pathophysiology of IPF involves the destruction of normal lung architecture along with inflammation and fibrosis. While the role of fibrosis is established in the pathogenesis of IPF, the role of inflammation remains controversial given multiple failed anti-inflammatory therapies as IPF treatment [4]. However, it has been hypothesized that chronic inflammation as a result of repeated injury to the lung epithelium partly due to the deposition of protein of the extracellular matrix (ECM) and dysregulated repair responses lead to eventual fibrosis [4,5]. 

**Figure 1:**
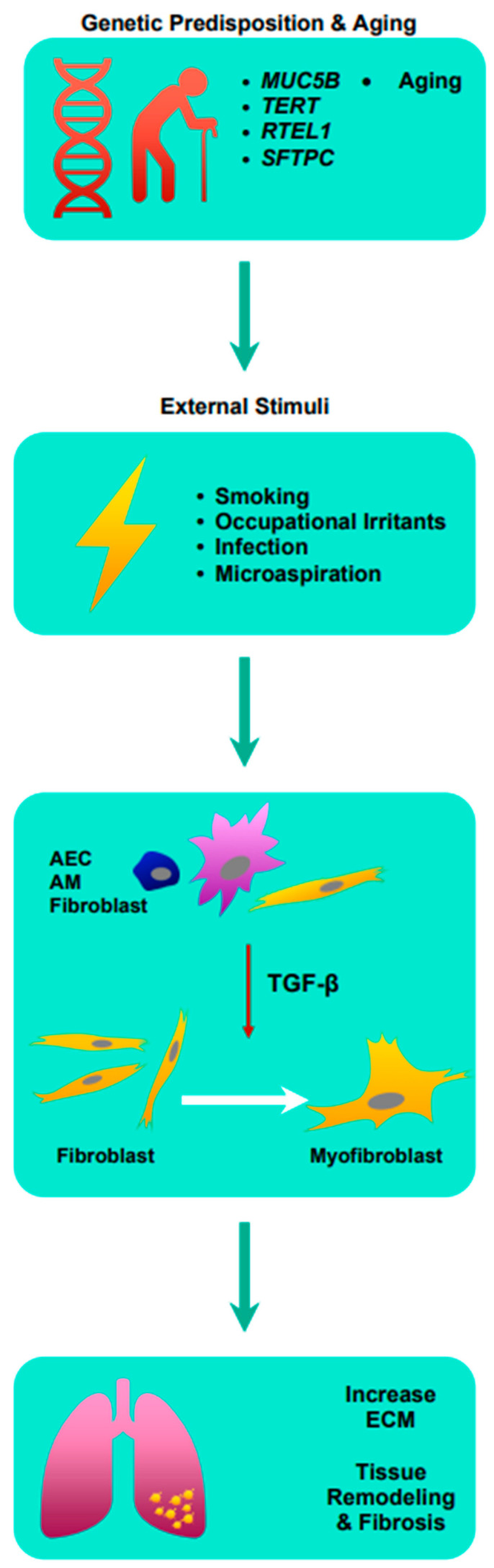
Summary of the pathophysiology of idiopathic pulmonary fibrosis. AEC: airway epithelial cell; AM: alveolar macrophages; ECM: extracellular matrix; MUC5B: Mucin 5B; RTEL1: regulator of telomere length 1; SFTPC: surfactant protein C; TERT: telomerase reverse transcriptase; TGF-β: transforming growth factor-β.

More recently, cytokine transforming growth factor-β (TGF-β) has been implicated in the development of pulmonary fibrosis [5,6]. TGF-β is pivotal in many cellular functions such as proliferation, differentiation, ECM synthesis, and apoptosis [7]. In the human lung, TGF-β is expressed by alveolar epithelial cells (AEC), alveolar macrophages, and fibroblasts, and is known to be elevated in cases of acute lung injuries, making it a critical mediator of inflammation [7,8,9,10]. Crucially, TGF-β induces the differentiation of myofibroblasts from fibroblasts, leading to the production of ECM such as collagen, laminin, and fibronectin [11]. Excessive ECM surrounding inflamed or injured lung tissue inhibits normal physiological functions of the cell including cell repair, and in cases of chronic inflammation, the balance in the production and degradation of ECM is affected due to increased TGF-β, consequently leading to ECM accumulation [12]. Subsequently, this leads to lung tissue remodeling and fibrosis. Understanding the pathophysiology of IPF is critical in the development of novel anti-fibrotics [See Figure 1].

Although the cause of IPF is largely unknown, significant milestones have been achieved in recent years with regard to the etiology of IPF. There are several genetic mutations and external factors that have now been identified that increases the risk of developing IPF. 

#### 1.1.1. Role of Genetic Mutation 

IPF, which occur in clusters in families, are referred to as familial interstitial pneumonia (FIP), a phenomenon that has been observed as early as 1950 [13]. There are now several genetic mutations that have been identified that play an important role in the development of IPF. A common polymorphism in the promoter of Mucin 5B, encoded by the gene MUC5B, is associated with both FIP and sporadic pulmonary fibrosis [14]. An intercontinental genetic study found that mutation in MUC5B is the strongest risk variant for IPF; higher with two copies of risk allele vs. one copy of risk allele with an odds ratio of 18.7 and 5.5, respectively [15]. Mucin 5B is a gel forming mucin that is essential in mucociliary clearance and is overexpressed in IPF lungs, leading to mucociliary dysfunction and increased fibrosis [16]. 

Short telomere syndrome, as a result of telomerase mutations, is another risk factor for FIP. It has been shown in in vitro studies that in the lung of an IPF patient with known telomerase mutation, shorter telomerases are seen in the alveolar epithelium compared to normal individuals [17]. Short telomeres reduce the tissue renewal capacity and telomeres shorten as an individual ages, which means that this could be the reason why the diagnosis of IPF has an age-related onset [17]. Moreover, cell senescence plays an essential role in resisting environmental stress and repopulating injured bronchoalveolar epithelia [15]. A very recent study involving over 120 centers globally studied 2180 cases of IPF using whole-genome sequencing to investigate the role of rare variants on IPF risk found that rare variants within the telomerase reverse transcriptase (TERT) and regulator of telomere length 1 (RTEL1) genes were significantly associated with IPF [18]. 

Genes encoding for surfactant protein C (SF-C) such as SFTPC have also been implicated in the diagnosis of FIP where a genetic study demonstrated, in a family of five generations, that individuals who were heterozygous for the mutation had biopsy changes consistent with usual interstitial pneumonitis (UIP) or non-specific interstitial pneumonitis (NSIP) [19]. Furthermore, SFTPC mutations have also been identified in sporadic cases of IPF [20]. In a normal mature lung, the pulmonary surfactant is secreted by type 2 AEC and functions by reducing surface tension in the alveolus and stabilizing the alveoli, maintaining lung volumes at end-expiration [21]. Mutations in SFTPC cause abnormal surfactant processing, leading to endoplasmic reticulum stress in type 2 AEC and eventual fibrosis of the lung tissue [22,23]. 

#### 1.1.2. Role of Environmental Factors

It has long been speculated that environmental factors or irritants such as cigarette smoke and occupational dust exposure could be a risk factor for developing IPF. Cigarette smoking has been the most studied of them all given its reputation in other chronic lung diseases. Experts believe that cigarette smoking may promote the transformation of lung fibroblasts to myoblasts, contribute to the shortening of the telomeres, and inducing endoplasmic reticulum stress [23,24,25]. A prospective study looking at over 800 individual IPF cases in the UK concluded that both active and maternal tobacco smoking increased the risk of IPF on top of having a synergistic effect with one another [26]. Furthermore, environmental factors may play an additive role to genetic factors contributing to the pathogenicity of IPF, where genetically susceptible lung tissue segments may experience repeated microscopic injuries from environmental irritants, eventually homogenizing into widespread fibrosis [15]. 

#### 1.1.3. Role of Infection

Some evidence suggests that the chronic infection of viruses such as EBV, CMV, HHV-7, and HHV-8 increases the risk of developing IPF [27,28]. The basis of this is immunosenescence, where persistent viral infection is thought to cause exhaustion of the immune system overtime, leading to T cell aging [29]. As aging is associated with IPF, some experts believe that immunosenescence is a risk factor for developing IPF [30]. More recently, SARS-Co-V2 infections was observed to increase pro-fibrotic macrophages, similar to those seen in IPF patients [31]. While at present the role of bacterial infection in the induction of IPF is unclear, it is thought to be more likely linked to the progression rather than initiation of fibrosis in patients [32,33]. 

#### 1.1.4. Aging and Cellular Senescence 

While genetic pre-disposition (i.e., mutations in telomeres) may lead to premature aging, physiological aging itself is a risk factor for developing IPF. Cellular senescence is a form of irreversible permanent cell cycle arrest and occurs naturally with aging, however, it may occur at any stage of an individual’s life [34]. The quantity of senescent cells increases with age as well as in aging-related diseases such as IPF, but the underlying mechanism that leads to physiological or pathological cellular senescence remains to be elucidated [35]. Senescence from normal aging on top of induced senescence from smoking, occupational irritants, or infection may accelerate cellular senescence. The implications of accelerated senescence are dysregulations of the innate and adaptive immune system including abnormal AEC and fibroblast activation as well as increased oxidative stress leading to impaired cell repair [36]. 

### 1.2. Diagnosis 

The diagnosis of IPF requires clinical co-relation with the presence of a typical pattern of usual interstitial pneumonia (UIP), identified either histologically or via computed topography [37]. Histopathological hallmarks of UIP are a combination of four features that are: (1) patchy dense fibrosis with architectural distortion; (2) predilection for subpleural or paraseptal lung parenchymal; (3) presence of fibroblastic foci; and (4) absence of features of alternative diagnoses [2]. Radiological features of UIP include the presence of honey combing with or without traction bronchiectasis, ground glass opacification, and interlobular septal thickening along with typical distribution in the subpleural or bases [2]. Based on the radiological and or histological features, the diagnosis of IPF is described as definite, likely, indeterminate, or alternate diagnosis suspected [2]. 

## 2. Anti-Fibrotic Agents

In the last decade, the emergence of two anti-fibrotic agents have been a major milestone in the treatment of IPF. Pirfenidone and nintedanib are the two licensed therapeutics in multiple jurisdictions used in the treatment of idiopathic pulmonary fibrosis. While these drugs significantly ameliorate the symptoms and survival in IPF patients, they are only able to slow down disease progression and not cure the disease [See Table 1]. 

### 2.1. Pirfenidone (Esbriet^®^)

Pirfenidone possesses both anti-fibrotic and anti-inflammatory effects [38]. The main proposed mechanism of action of pirfenidone’s anti-fibrotic effect is mediated through the inhibition (expression and activity) of TGF-β at a transcriptional level [38,39]. Consequently, this reduces the differentiation of fibroblasts, myofibroblasts, collagen, fibronectin synthesis, and the deposition of ECM [40]. On the other hand, pirfenidone blocks the production of inflammatory cytokines such as tumor necrosis factor alpha (TNF-α), interleukin-1 (IL-1), IL-4, and IL-13, leading to an overall anti-inflammatory effect [41,42]. Additionally, the anti-inflammatory effect is also modulated through the inhibition of the formation of a pro-inflammatory protein complex, the NLRP3 inflammasome [43,44].

Pirfenidone is rapidly absorbed via the oral route and binds to serum albumin at a mean rate of approximately of 50–58% in clinically studied serum concentrations (1 ug to 100 ug/mL) [45]. Pirfenidone is primarily excreted through the renal route with approximately 87% of the orally administered drug being excreted in urine in the form of the primary metabolite, 5-carboxy-pirfenidone [46]. In mild renal or hepatic impairment, dose adjustment is not necessary, however, pirfenidone is contraindicated in severe renal and hepatic impairment [45]. Pirfenidone possesses photoreactive properties, which leads to the generation of reactive oxygen species (ROS) upon contact with UV rays, and for this reason, may cause photosensitivity of the skin [47]. Hence, patients on pirfenidone are advised to minimize their exposure to direct sunlight by applying sunblock daily or to wear protective clothing against sun exposure to reduce the risk of photosensitivity [45].

A variety of trials have demonstrated the efficacy of pirfenidone in the reduction of disease progression. One of the first major trials, the CAPACITY trial, which consisted of two concurrent trials (004 and 006), involved a total of almost 800 patients randomized to receive either pirfenidone 2403 mg/day, pirfenidone 1197 mg/day, or the placebo over 72 weeks with the primary endpoint being change in forced vital capacity (FVC) at the end of week 72. There was a consistent effect in the reduction of FVC at 48 weeks in the pirfenidone arm, however, the FVC change at week 72 was not significant in study 006 [48]. The findings of this study were overall encouraging, and this was followed by the ASCEND trial a few years later with 555 participants randomized to receive either 2403 mg of pirfenidone or placebo over the course of 52 weeks. The study demonstrated that there was nearly a 50% reduction in the proportion of patients who had an absolute decline of 10% in the predicted FVC or those that died, the primary endpoints [49]. Pirfenidone also demonstrated efficacy in these trials’ secondary endpoints: reduced decline in 6-min walk test (*p* = 0.04) and improved progression free survival (*p* < 0.001) [48,49]. Most common side effects in the pirfenidone group from the ASCEND trial was nausea (36.0%) and rash (28.1%), similar to what was seen in the CAPACITY trial (nausea 36.0% and rash 32.0%) [48,49]. 

### 2.2. Nintedanib (Ofev^®^)

Nintedanib is a tyrosine kinase inhibitor, and its mechanism of action is achieved through the deactivation of tyrosine kinase receptors, more specifically, platelet derived growth factor-receptor (PDGFR), fibroblast growth factor receptor (FGFR), and vascular endothelial growth factor receptor (VEGFR) [50]. The corresponding ligands of these receptors (i.e., PDGF, FGF, and VEGF) enhance fibroblast migration and proliferation in the lung, leading to pulmonary fibrosis [51]. Nintedanib binds to the ATP binding pocket of these receptors, preventing auto phosphorylation and thereby inhibiting downstream cascade signaling [50]. 

Upon the administration of nintedanib, the compound undergoes hydrolytic ester cleavage, producing a free acid moiety that is then further metabolized via glucuronidation and almost entirely excreted in the feces [52]. Nintedanib is approximately 98% plasma protein bound and has a bioavailability of approximately 4.7% and orally, the maximum plasma level of nintedanib is achieved at 2 to 4 h with a half-life of 10–15 h [52]. Although nintedanib is a minor substrate for cytochrome p450 (CYP), drug–drug interventions with other CYP inhibitors or inducers are unlikely to affect its efficacy [52]. However, a dose reduction is recommended in mild hepatic impairment (Child–Pugh A) and not recommended in moderate or severe hepatic impairment [53]. 

Two large trials (INPULSIS 1 and 2) examined the efficacy of nintedanib by comparing the annual decline in FVC with a placebo group over the course of one year. INPULSIS-1 demonstrated a decline of 114.7 mL per year in the nintedanib group and 239.9 mL per year in the placebo group (*p* < 0.0001). Similarly, INPULSIS-2 showed a rate of decline in FVC of 113.6 mL per year in the nintedanib group and 207.3 mL per year in the placebo group (*p* = 0.0002). These results were consistent amongst the pooled data from the two INPULSIS trials, covering a range of pre-specified subgroups defined by sex, age (<65 or ≥65 years), race (White or Asian), baseline FVC predicted % (≤70% or >70%), baseline SGRQ total score (St George’s Respiratory Questionnaire Score ≤40 or >40), smoking status (never smoked or current/ex-smoker), and systemic corticosteroids at baseline (yes or no). Two secondary endpoints within these trails examined the time to first exacerbation and reduction in SGRQ. It was noted in INPULSIS-2 that the proportion of patients with at least one exacerbation was lower in the nintedanib group than in the placebo group (3.9% vs. 9.6%), hazard ratio of 0.38 (*p* = 0.005); however, no significant difference was noted in INPULSIS-1. The mean change in the baseline in the SGRQ total score at week 52 was −2.69 points (*p* < 0.02) in INPULSIS-2 and −0.05 points (*p* = 0.97) in INPULSIS-1 (lower scores indicate better functioning). In addition, sensitivity analysis on pooled data from both trials demonstrated a significantly reduced risk of time to first exacerbation (confirmed or suspected) in the nintedanib group compared to the placebo (HR 0.32; *p* = 0.001) [54]. Most common side effects in the nintedanib group from both INPULSIS-1 and INPULSIS-2 was diarrhea (61.5% and 63.2%) and nausea (22.7% and 26.1%) [54]. 

### 2.3. Combination Pirfenidone (Esbriet^®^) and Nintedanib (Ofev^®^)

As there are various pathways involved in the development of IPF, it is possible that providing multiple therapies to target different pathways offer synergistic effects [55]. More recently, there has been growing interest in combining pirfenidone and nintedanib for the treatment of IPF. Pirfenidone, being a TGF-β inhibitor, abrogates the transcription of TGF-β, and nintedanib, inhibiting PDGF, FGF, and VEGF, reduces the migration of the fibroblast, overall leading to a decrease in the accumulation of myofibroblasts. 

Safety and tolerability studies investigating the combination therapy of pirfenidone and nintedanib have been promising, where combination therapy was similarly tolerated with a manageable safety profile compared to monotherapy [56,57,58]. The INJOURNEY trial, an open labelled randomized trial compared the safety and efficacy of monotherapy nintedanib (150 mg twice daily) vs. combination nintedanib (150 mg twice daily) and pirfenidone (titrated to 801 mg three times daily) over 12 weeks in IPF patients with low to moderate lung function impairment after 4 to 5 weeks of run-in with nintedanib at full dose. The authors found no increase in the plasma level of nintedanib or adverse events in combination therapy compared to monotherapy, on top of an observation of about a 50% reduction in the rate of decline in lung function (mean change in FVC: −13.3 mL in combination therapy vs. −40.9 mL nintedanib alone). Reported adverse events (any) were 88.7% in the combination treatment group and 88.2% in the monotherapy group [57]. However, as the trial was only 12 weeks in duration, caution should be placed when attempting to draw any conclusions from an efficacy perspective. While the results from this study seem encouraging, further trials with longer time points need to be conducted to assess the efficacy of combination therapy over a longer duration of time. 

**Table 1:** Summary of the main clinical trials and their findings evaluating pirfenidone and nintedanib in IPF patients.

Drug	Trial(s)	Year Published	Study Design	Main Findings
Pirfenidone Esbriet^®^ (TGF-β inhibitor)	CAPACITY [48]	2011	72-week, multicenter randomized, double blind, and placebo controlled	A significant reduction in FVC was seen in the treatment group compared to the placebo. The treatment group had higher side effects; top three being nausea, dyspepsia, and vomiting.
	ASCEND [49]	2014	52-week, multicenter randomized, double blind, and placebo controlled	Pirfenidone reduced decline in FVC and 6-min walk distance. GI and skin-related adverse events more common in patients on pirfenidone.
Nintedanib Ofev^®^ (Tyrosine Kinase Inhibitor)	INPULSIS-1 & -2 [54]	2014	52-week, multicenter randomized, double blind, and placebo controlled	Nintedanib reduced the decline in FVC. Nintedanib reduced the time to first exacerbation in INPULSIS-2. Diarrhea was the most common side effect of nintedanib.
Pirfenidone plus nintedanib	INJOURNEY [57]	2018	12-week, open-label, randomized trial	No increase in adverse events in combination therapy. Reduced rate of decline in lung function in combination therapy compared to monotherapy.

FVC: forced vital capacity; GI: gastrointestinal; IPF: idiopathic pulmonary fibrosis; TGF-β: transforming growth factor-β.

## 3. Novel Treatment

While the current licensed anti-fibrotics pirfenidone and nintedanib significantly reduce disease progression, there is no treatment that stops progression or reverses the decline in lung function. Furthermore, issues regarding tolerability to an anti-fibrotic agent by a patient leave very limited options for dose titration and alternatives. There are now a growing number of compounds under investigation with ongoing clinical trials whose primary function is to serve as an alternative, replacement, or add on therapy, perhaps even halting progression or potentially reversing fibrosis [See Table 2]. 

### 3.1. Pamrevlumab

Pamrevlumab is a recombinant human antibody that binds to connective tissue growth factor (CTGF), which prevents the activation of downstream pro-fibrotic signaling [59]. CTFG is a protein that modulates signaling pathways in myofibroblast activation, ECM deposition, and remodeling [60]. It is the primary mediator of TGF-β and is overexpressed in areas of fibrotic lesions in major organs including the lungs of IPF patients [61,62,63]. In 2019, the phase 2 PRAISE trial demonstrated that intravenous pamrevlumab successfully decreased the decline in FVC by approximately 70% in IPF patients at 48 weeks compared to the placebo group [64]. Compellingly, the treatment benefit was apparent regardless of whether the FVC change was expressed as the change in percentage predicted values, change in volume, or as the majority component in a categorical analysis of progression-free survival, unlike in many other recent trials [65]. While the results should be treated with caution pending an adequately powered phase 3 study, the treatment effect was apparent with similar treatment effects in radiological (quantitative lung fibrosis HRCT scores) and symptoms (SGRQ score) and symptoms parameters, which has not been seen in other studies [64]. The results of this study are likely the most encouraging of all phase 2 trials and there are questions raised as to whether the treatment benefits of pamrevlumab might be amplified in combination with anti-fibrotic therapy [59,65]. 

### 3.2. PRM 151 

Pentraxin-2 (PTX2), also known as serum amyloid P, is a member of the Pentraxin protein family, constitutively expressed with established activity in modulating wound healing and the fibrotic remodeling of injured tissue [66]. Previous studies have demonstrated that circulating levels are decreased in fibrotic disease [67]. On a molecular level, PTX2 appears to modify neutrophil adhesion and inhibit the differentiation of monocytes into pro fibrotic macrophages and fibrocytes [68,69]. PTX2 is also postulated to inhibit the expression of TGF-B, the central fibrosis mediator [70]. In genetically modified murine models following bleomycin treatment, prolonged lung inflammation and increased fibrosis were noted in mice with deletion of the PTX2 gene [71]. 

A multicenter, randomized, phase 2 double blind study investigated recombinant human PTX2 protein (PRM-151) involving a patient cohort of 117 with IPF (ages 40–80) where participants were randomized to either a placebo or 10 mg/kg of PRM-151 every 4 weeks following a three-dose loading regimen and were stratified by concurrent IPF treatment status [72]. In the preliminary phase of the study, there was a 95.7% completion rate at 28 weeks and 111 patients moved onto the open-label crossover extension phase of the study [73]. In the PRM-151 treatment group, there was a reduction in decline in the percentage mean predicted FVC (−2.5 vs. −4.8) and 6-min walk distance in meters (−0.5 vs. −31.8), and this effect was sustained up to 52 weeks in patients who continued the treatment (predicted FVC decline of −3.6% and 6-min walk distance −10.5 m) [72,73]. In patients who commenced PRM 151 during the extension phase of the study, they had an improvement in percentage FVC decline from −8.7% per year to −0.9% per year and 6-min walk distance from −54.9 m per year to −3.5 m per year. About 28% of patients experienced an adverse event, however, they were consistent with the long-term complications of IPF [73]. 

### 3.3. GLPG 1690

Another drug currently being trialed is GLPG 1690, a potent and selective autotaxin inhibitor [74]. Autotaxin is a secreted lysophospholipase D involved in extracellular lysophosphatidic acid production. Autotaxin is elevated in many inflammatory and fibroproliferative conditions, which in turn increases lysophosphatidic acid, activating various pro-inflammatory signals including TGF-β and causes fibroblast accumulation [75]. A phase 2a randomized placebo-controlled trial (FLORA) resulted in an observed improvement in FVC in the treated patients (25 mL) vs. the placebo (−70 mL) at week 12, encouraging further development of GLPG1690 as a potential novel treatment for IPF patients [74]. The trial was only 12 weeks in duration, hence it was not sufficient to investigate the long-term efficacy of GLPG1690. There are currently two phase 3 trials ongoing. 

### 3.4. BI 1015550

Oral phosphodiesterase type 4B compound (BI 1015550) is also currently in its clinical trial phase. These agents enhance the adenosine 3′,5′-cyclic monophosphate (cAMP) levels (through inhibition of hydrolysis), which in turn is vital to the function of prostaglandin E2, implicated in the inhibition of all relevant functions of fibroblasts [76]. BI 1015550 blocks mitogen induced fibroblast proliferation and appears to effectively work in tandem with nintedanib to inhibit this response, demonstrating a ten-fold shift to the left in the dose–response curve. This compound also appears to inhibit TGF β1–induced myofibroblast transformation and the mRNA mediated expression of certain ECM proteins, which gives it a therapeutic advantage over nintedanib [77]. 

A phase 2 double-blind, placebo-controlled trial recruited 147 patients aged over 40 with an existing diagnosis of IPF with or without background antifibrotic treatment and evaluated the efficacy of BI 1015550 over a 12-week period with the primary endpoint being a change in baseline forced vital capacity (FVC) [78]. Interestingly, in patients without pre-existing antifibrotic use, the median change in FVC was 5.7 mL (95% CI, −39.1 to 50.5) in the BI 1015550 group and −81.7 mL in the placebo group (95% CI, –133.5 to –44.8) whereas in patients with pre-existing anti-fibrotic use, the median change was 2.7 mL (95% credible interval, –32.8 to 38.2) in the BI 1015550 group and −59.2 mL in the placebo group (95% credible interval, –111.8 to –17.9). The calculated probabilities that BI 1015550 was superior to the placebo in each group were 0.998 and 0.986, respectively. Diarrhea was the most common side effect that led to the discontinuation in 13 patients (10 were on background anti-fibrotics [78]). Caution is required when interpretating the efficacy of BI 1015550 in this study as the treatment duration was short and the sample size small. A phase 3 trial is currently in its recruiting phase. 

### 3.5. PBI 4050

G protein-coupled receptors with free fatty acid ligands such as G protein receptor (GPR)40 and GPR84 have been implicated in inflammatory conditions including being modulators of fibrosis [79]. GPR40 is expressed in epithelial cells while GPR82 is in the immune cells, and activation of both GPR40 and GPR82 reduces fibrosis via the regulation of macrophages, fibroblasts, myoblasts, and epithelial cells [79]. PBI-4050 (3-pentylbenzeneacetic acid sodium salt) is an orally active synthetic analogue of a medium-chain fatty acid that has agonist and antagonist ligand effects toward GPR40 and GPR84, respectively [79]. 

The phase 2 clinical trial of PBI-4050 in IPF patients showed that when used alone or in combination with nintedanib or pirfenidone, it was well-tolerated. Moreover, the authors reported the stability of FVC at week 12 in patients on PBI-4050 and combination PBI-4050 plus nintedanib [80]. It is to be noted, however, that the trial was not blinded, hence, there were no placebo control group. 

### 3.6. PLN 74809

Integrins are heterodimer transmembrane proteins and there is strong evidence that they are closely related to TGF-β production [81]. In fibrotic lungs, αv integrins are expressed at high levels by epithelial cells and fibroblasts [82]. Furthermore, in in vitro models, the dual inhibition of αvβ6 and αvβ1 has been shown in decrease fibrosis in both murine and human lung tissue [82]. PLN 74809 is an oral small molecule inhibitor of integrins αvβ6 and αvβ1 and the inhibition of these molecules may offer a novel localized means of suppressing TGF-β in fibrotic lung tissue and potentially reducing the systemic side effects in the treatment of idiopathic pulmonary fibrosis [82]. 

PLN 74809 is currently being evaluated in a phase 2a, open, 4-part, randomized, double-blind, dose-ranging (60–320 mg), placebo-controlled study to evaluate the safety, tolerability, and pharmacokinetics in participants with IPF (INTEGRIS-IPF). The 12-week preliminary results of this trial were presented at the European Respiratory Society (ERS) and American Thoracic Society (ATS) conference in 2020 and 2021, respectively [83,84]. The study had approximately 90 participants, over 40 years old, with IPF. The primary endpoints were the safety, tolerability, and pharmacokinetics of PLN 74809 and the exploratory endpoints measured the change in FVC, quantitative lung fibrosis score (QLF, as evaluated by high resolution computerized tomography, HRCT), cough symptoms via the visual analogue scale, and applicable biomarkers over a 12-week period. Pliant therapeutics has since released the pre-liminary results (prior to peer reviewed publication): PLN 74809 demonstrated good efficacy, safety, and tolerability. The average decline in FVC for patients on the placebo was 74.1 mL and for all patients on PLN-74809, it was 15.1 mL. In terms of efficacy with respect to dose range, the average FVC decline was 38% lower in the 40 mg dose group and 66% lower in the 160 mg dose group. Most notably, the proportion of those with a predicted 10% (or more) reduction in FVC (an independent risk factor for disease progression and death in IPF) was 17.1% for the placebo and 18.2%, 8.7%, and 4.5% for the 40 mg, 80 mg, and 160 mg groups, respectively. The average change in QLF was 1.15% in the placebo group compared with 3.15%, 0.70%, and 0.00% in the 40, 80, and 160 mg groups, respectively. Higher QLF scores indicate more fibrosis, as evaluated by imaging (HRCT), demonstrating that those on dosages of 80 mg and above showed either stable or improved scores, similar results to the dose range for the proportion of those with a predicted 10% or more reduction in FVC [85].

In keeping with the promising results demonstrated to date for higher dosages, Pliant has announced an extension to this trial investigating the efficacy of 320 mg per day doses of PLN-74809 over the course of 6 months in participants with IPF. Preliminary results from this part of the trial should be available in early 2023 [85].

### 3.7. BMS 986020

Lysophosphatidic acid receptor-1 (LPA1) has been shown in murine models to regulate fibrosis via mediation of the fibroblast recruitment and vascular leakage on top of being elevated in patients with IPF [86]. The LPA1 antagonist BMS-986020 was evaluated in a phase 2, parallel-arm, multicenter, randomized, double-blind, placebo-controlled trial over 26 weeks involving 143 patients randomized to receive BMS-986020 600 mg OD, 600 mg BID, and the placebo [87]. It was found by the authors that there was a significant reduction in the rate of decline in the FVC between the placebo group vs. BID dosage group but not the OD at the end of 26 weeks. Unfortunately, adverse reactions of hepatobiliary toxicity (elevated liver enzymes) and treatment-related cholecystitis in a number of patients led to early termination of the study.

### 3.8. TD 139

Galectin (Gal)-3 is a glycan binding protein with pleotropic effects involving cellular communication, inflammation, development, and differentiation [88]. Gal-3 has been shown to be a potent regulator of the fibrotic processes and is increased in the BAL fluid of patients with IPF [89], which makes it a natural choice as a potential therapeutic target. TD 139 is a small molecule inhibitor of Gal-3 thought to exert its effect through the high affinity binding of the carbohydrate recognition domain of Gal-3 [90]. 

The efficacy of TD 139 was evaluated through a randomized, multicenter, placebo controlled, phase 1/2a UK based trial where 60 participants were recruited (36 healthy and 24 with a diagnosis of IPF) and divided into dose cohorts and administered single doses of TD 139 ranging from 0.15 to 50 mg over the course of 14 days [90]. The primary endpoint of this trial evaluated the safety and tolerability of single or multiple doses of TD139 in healthy vs. IPF patients and secondary endpoints included pharmacokinetic analysis, expression of Gal-3 in the lung and blood, and phenotypic analysis of lung macrophages in addition to plasma biomarkers following treatment with TD139. Pharmacokinetic parameters were largely similar between the healthy and IPF participants apart from TD139 being less retained in the lungs in IPF patients. Moreover, TD139 was reported to be well-tolerated by both healthy and IPF patients with taste disturbance (36.1%), and cough (11.1%) being the most common side effects, and no clinically significant changes noted in any biochemical, hematological markers, electrocardiographs, or clinical observations [90].

### 3.9. Inhaled Sodium Cromoglycate (New Formulations) 

Novel formulations of inhaled sodium cromoglycate that can be delivered via nebulizers have been trialed in treating chronic cough in patients with IPF [91]. Sodium cromoglycate is a mast-cell stabilizer; topical and oral formulations have been used to treat mastocytosis and older inhaled formulations are currently approved for the treatment of asthma [92,93]. The novel formulations of sodium cromoglycate allow the drug to be delivered more efficiently with higher lung deposition, unlike the traditional inhaled devices [91]. 

A randomized, double-blind, proof-of-concept, cross-over, phase 2 trial looked at 33 patients with IPF on anti-fibrotics and treated them with PA101 (novel formulation of sodium cromoglycate) for 2 weeks and assessed their cough frequency and severity thereafter. At the baseline, there was no difference in cough frequency and severity between the treatment group and placebo group, however, there was a mean reduction in daytime cough frequency at day 14 (31.1%) and at 24 h (29.1%) when adjusted for the placebo [91]. Side effects experienced by the participants included headache, diarrhea, dry mouth, and flushing, however, they were mild to moderate in nature and there was no significant difference between the treatment and placebo group [91]. Another phase 2b trial (SCENIC) that investigated varying doses of inhaled sodium cromoglycate over 12 weeks in IPF patients found no benefit over the placebo in reducing cough [94]. However, due to the COVID-19 pandemic, the recruitment process was incomplete and had to be prematurely terminated. 

### 3.10. TRK 250 

TRK 250, previously known as BNC 1021, is a novel agent that consists of a single stranded oligonucleotide capable of producing silencing RNA (siRNA) targeting TGF-β1 mRNA. In essence, TRK 250 inhibits the transcription of TGF-β1 [95]. This drug is developed by Toray Industries, Inc. and is expected to be in the form of an aerosol, hence it can be administered directly to the lung [96]. TRK 250 has demonstrated its ability to reduce the expression of TGF-β1 and collagen production in the lungs in animal models [95]. A phase 1 placebo-controlled, double-blind, randomized study assessing the safety and tolerability of single and multiple inhaled doses of TRK 250 in subjects with IPF for 4 weeks was completed in April 2022 [97]. However, the results of the study are yet to be released.

### 3.11. Dasatinib (D) + Quercetin (Q)

As IPF is now established as an aging-related disease, targeting senescent cells as a therapeutic strategy is appealing to many experts in the field of immunosenescence. Dasatinib (D), a tyrosine kinase inhibitor, and quercetin (Q), a flavonoid, used in combination (DQ) has previously been demonstrated to be an effective senolytic agent in vitro in human and murine cells [98,99,100]. DQ possesses the ability to induce selective apoptosis in senescent cells within 48 h and possibly prevent the physical dysfunction and accelerated onset of age-related diseases [101]. 

The first human open-label, pilot study examining this combination drug by administering intermittent D (100 mg/day) plus Q (1250 mg/day) orally over three consecutive days in three consecutive weeks in patients with IPF was conducted in 2016 with the main aim to investigate the feasibility of administrating senolytic therapy [102]. The primary endpoint was the retention rates and completion rates for planned clinical assessments. Secondary endpoints were safety and change in functional and reported health measures. The study recruited 14 patients in total and achieved 100% retention rates. One severe adverse event (SAE) occurred where the patient developed multi-focal bacteria pneumonia that resulted in hospitalization. The most common side effects were skin irritation and GI discomfort. While there was significant improvement in the 6-min walk distance (*p* < 0.05), there were no improvements in the FVC, frailty index (FI-LAB), and reported health [102]. Although the study had a small sample size, was short in duration, and unblinded, this is the first of its kind and highlights the potentials of repurposing old drugs by targeting novel pathways to treat IPF.

**Table 2:** Summary of the clinical trials and their main findings for novel therapies in IPF patients.

Drug	Trial	Year Published	Study Design	Main Findings and Follow Up
Pamrevlumab (Recombinant human antibody)	PRAISE [64]	2020	48-week phase 2, randomized, double-blind, placebo-controlled	Reduction of decline in FVC at 48 weeks seen in pamrevlumab group compared to placebo. No significant side effects seen from pamrevlumab. Phase 3 trial currently ongoing. NCT03955146.
PRM 151 (Recombinant human PTX2 protein)	NCT02550873 [72,73]	2018 2019	28-week phase 2 double-blind, randomized controlled trial 76-week open-label crossover extension study	Improvement in decline of % FVC and 6-min walk distance. No significant side effects from the drug. Phase 3 trial currently ongoing. (STARSCAPE; NCT04552899).
GLPG 1690 (autotaxin inhibitor)	FLORA [74]	2018	12-week phase2, multicenter, randomized, double-blind, placebo-controlled	GLPG1690 had no difference in adverse reactions compared to placebo group Observed improvement of FVC in treatment group compared vs. placebo. Two phase 3 trials ongoing (ISABELA-1, and 2). NCT03711162 NCT03733444.
BI 1015550 (PDE type 4B compound)	NCT04419506 [78]	2022	12-week phase 2 double-blind, placebo-controlled, parallel-design.	Patients treated with BI 1015550 had reduction in FVC decline regardless of other background anti-fibrotic use. Most common side effects of BI 1015550 was GI related symptoms. Phase 3 trial in recruiting phase NCT05321069.
PBI 4050 (G-Protein Receptor analogue)	NCT02538536 [80]	2019	12-week phase 2 single-arm open-label study.	PBI 4050 monotherapy or combination with either nintedanib or pirfenidone was well-tolerated. Altered pharmacokinetics seen in PBI 4050 plus pirfenidone but not nintedanib suggesting drug-drug interaction. FVC stability at week 12 in PBI 4050 and combination PBI and nintedanib. Phase 3 trial planned.
PLN 74809 (Integrins αvβ6 and αvβ1 inhibitor)	INTEGRIS-IPF [85]	2023 (expected)	12-week phase 2, multicenter, randomized, double-blind, placebo-controlled	Preliminary results presented at the ERS and ATS conference in 2020 and 2021 Preliminary results released by Pliant Therapeutics: No significant SAE noted with PLN 74809. Reduction in decline in FVC seen in the PLN 74809 group in a dose dependent manner. Plan for extension of trial investigating the efficacy of 320 mg per day over 6 months. NCT04396756.
BMS 986020 (Lysophosphatidic acid receptor-1 inhibitor)	NCT01766817 [87]	2018	26-week phase 2 randomized, double-blind, placebo-controlled.	BMS 986020 significantly reduced the decline in FVC compared to placebo. Early termination of study due to SAE (Cholecystitis).
TD139 (Inhaled galectin 3 inhibitor)	NCT02257177 [90]	2021	2-week phase 1/2a randomized, double-blind, multicenter, placebo-controlled.	Inhaled TD139 is safe and well tolerated in healthy subjects and IPF patients Gal-3 expression in alveolar macrophages in treatment group was lower compared to placebo (dose dependent) Phase 2b study now ongoing (GALACTIC-1; NCT03832946)
PA 101 (Inhaled sodium cromoglycate)	NCT02412020 [91]	2017	2-week pilot, proof-of-concept study, randomized, double-blind, placebo-controlled.	PA101 reduced daytime cough in IPF at day 14 compared to the placebo. No SAE observed. A phase 2b trial (SCENIC) was terminated in 2020; NCT03864328.
TRK 250 (siRNA-Based Oligonucleotide)	NCT03727802 [97]	NA	4-week phase 1 randomized, double-blind, placebo-controlled.	Results awaited NCT03727802.
Dasatinib/Quercetin; DQ (Tyrosine Kinase inhibitor/flavonoid)	NCT02874989 [102]	2019	3-week pilot, open label study	100% completion rate achieved. Most common side effects: skin irritation and GI discomfort. Improvement in 6-min walk distance but no improvement in FVC. No further trials planned at present.

FVC: forced vital capacity; GI: gastrointestinal; SAE: severe adverse event.

## 4. Adjunctive Treatment

Prior to the emergence of anti-fibrotic therapy, there were numerous drugs that were trialed, most resulting in disappointing results. However, as older drugs are more economical and accessible, there is ongoing interest in investigating the efficacy of these drugs in IPF patients either as a monotherapy or in combination (adjunctive) with anti-fibrotic therapy. 

### 4.1. N-Acetylcysteine (NAC) 

N-acetylcysteine (NAC) was one of the first few drugs trialed for the treatment of IPF prior to the emergence of anti-fibrotic therapies. NAC is well-known for its antioxidant properties and has been used widely for a very long time in cases of acetaminophen overdose. The main mechanism of action of NAC is by providing a precursor of cysteine, which is required in the production of glutathione (GSH), a potent antioxidant of hydrogen peroxide. Administering NAC in paracetamol poisoning rapidly increases the production of GSH, which increases the clearance of acetaminophen from the system. NAC was first implicated in IPF when it was observed that alveolar structures of IPF patients had inflammatory cell-mediated oxidant injury [103]. Furthermore, it was noted that in patients with IPF, there was a deficiency in GSH in the lung epithelium, suggesting an oxidant–antioxidant imbalance [104]. 

In 2005, a double-blind, randomized, placebo-controlled multicentered study assessed the effectiveness of a high oral dose of NAC (600 mg three times daily) added to standard therapy with prednisone and azathioprine over 1 year. The authors concluded that the addition of NAC to the standard therapy of prednisolone and azathioprine had better preserved vital capacity and DLCO [105]. This was followed by a high-profile study (PANTHER-IPF) in 2012, where the IPF patients with mild to moderate lung function impairment were assigned to one of three groups receiving a combination of prednisolone, azathioprine, plus NAC, NAC alone, or placebo at an equal ratio to all groups. There was an increased risk of death seen in the combination treatment group compared to the monotherapy and placebo group, resulting in the early termination of the combination treatment group [106]. The same research group later reported on the results of monotherapy NAC and the placebo. Unfortunately, the authors found that NAC offered no significant benefits in preserving FVC in patients with mild to moderate impairment of lung function compared to the placebo [107]. A post hoc secondary analysis of the trial postulated that an individual’s response to NAC may be predicted by their genetic mutation with favorability toward those with MUC5B mutations [108]. Nevertheless, there have since been several smaller studies that have explored inhaled NAC as a combination therapy or monotherapy in IPF patients. However, evidence is still insufficient for its use in clinical practice [109,110,111]. 

### 4.2. Corticosteroids

Corticosteroids are a group of steroid hormones that possess potent anti-inflammatory properties. Corticosteroids are divided into glucocorticoids and mineralocorticoids that bind to the glucocorticoid receptor (GR) and mineralocorticoid receptor (MR), respectively. The anti-inflammatory property is achieved through the GR receptor by reducing the expression of transcription factors activator protein-1 (AP-1) and NFκB, which subsequently reduces the production of pro-inflammatory cytokines such as IL-1β [112]. Prior to the emergence of disease modifying agents for IPF, the treatment of IPF has mainly focused on reducing the inflammation that presents with the disease. Utilizing the anti-inflammatory properties of corticosteroids as a treatment itself for IPF have been studied since the 1980s, however, studies have not demonstrated any promising results [113,114,115]. This includes the use of prednisolone as an adjunctive treatment in the PANTHER-IPF trial, which did not result in any improvement in the clinical outcomes [106]. In 2000, the American Thoracic Society released an internal consensus statement concluding that based on evidence available at that time, it is unlikely that corticosteroids offer any cure for the disease, but prolonged treatment of 1 to 2 years may be reasonable for patients who are responders [116]. In 2011, the recommendations were revised to strongly recommend against the use of corticosteroid monotherapy in the treatment of IPF [37]. Presently, corticosteroids are mainly used by clinicians at a discretionary basis in the acute exacerbation of IPF as part of supportive treatment [2,117]. 

### 4.3. Antacids

It has been postulated by many clinicians and experts that gastroesophageal reflux disease (GERD) may be related to IPF in relation to disease progression, and perhaps development. Some have hypothesized that chronic silent micro-aspiration of acidic content from GERD leads to the pathogenesis of some IPF [118]. Interestingly, it has been previously demonstrated in in vitro studies that exposure of lung epithelial cells to bile acids (chenodeoxycholic acid and glycochenodeoxycholic acid) that are frequently associated with GERD causes an increased expression of TGF-β and fibroblast proliferation [119]. However, a meta-analysis that included 18 case-control studies looking at the association of GERD and IPF concluded that there may be an association, but that it is likely that the association is confounded by various factors, especially smoking [120]. Nevertheless, GERD treatment is often offered as the prevalence of GERD in IPF patients is not uncommon and may be as high as 87% [121,122]. A pooled analysis conducted in 2013 looked at three clinical trials that reported that IPF patients on either a proton pump inhibitor or histamine receptor 2 (H2) blocker had a smaller decrease in FVC at 30 weeks [123]. More recent studies, however, have shown that IPF patients on antacid therapy or those who had anti-reflux surgery did not have better outcomes than those with who did not [124,125,126]. Hence, the current guidelines recommend against the use of antacid medications and anti-reflux surgery in IPF patients for the purpose of improving lung function [2]. 

### 4.4. Azithromycin 

One of the more prominent symptoms of IPF is debilitating cough. IPF patients who never smoked and have more advance disease are more likely to experience cough, and is an independent predictor of disease progression, time to death, or lung transplantation [127]. While anti-fibrotics can reduce the symptoms of cough to a certain extent, cough may persist in some patients [128,129]. Azithromycin, an antibiotic with known immunomodulatory effects, has been shown to be successful in the treatment of chronic cough in COPD patients [130]. A double-blind randomized controlled crossover study trialed prophylactic azithromycin at 500 mg three times per week or the placebo three times a week to assess the safety and efficacy of azithromycin for the treatment of chronic cough in IPF patients yielded disappointing results [131]. 

### 4.5. Co-Trimoxazole

As there has been increasing interest in lung microbiome in the initiation, progression, and exacerbation of fibrosis in IPF patients [32,33], the potential of co-trimoxazole, a sulfonamide antibiotic as prophylaxis or adjunct therapy has garnered some attention. In a multicenter double blinded study of 181 patients, co-trimoxazole, in addition to standard therapy, increased the quality of life and reduction in mortality but had no effect on lung function [132]. Based on the promising results of this study, the EME-TIPAC trial emerged; a double-blind, placebo-controlled, randomized, multicenter clinical trial based in the UK intending to look at the efficacy of co-trimoxazole in patients with moderate to severe IPF. The results were unfortunately disappointing, showing that treatment with co-trimoxazole on top of standard therapy did not reduce the outcome of time to death, transplant, or non-elective hospitalization compared with the placebo [133].

### 4.6. Anti-Viral Therapy

The role of viral infections in the pathogenesis of IPF is unclear at present, but CMV, EBV, and herpesvirus have been found to be more frequently present in IPF lungs compared to healthy controls [22,134]. Infection with herpesvirus is thought to cause altered surfactant protein processing in AEC, leading to endoplasmic reticulum stress, and consequently fibrosis and disease progression [22]. A phase 1 randomized controlled clinical trial reported that valganciclovir was well-tolerated as an add on to anti-fibrotic pirfenidone in patients with IPF [135]. There was an observed trend toward improved FVC at week 12 in the valganciclovir treated group, however, as the numbers were small and the duration was short, a conclusion cannot be drawn regarding the effects on lung function, especially on a long-term basis. 

Another anti-viral agent, ganciclovir, has also been studied. An observational study that looked at advanced IPF patients with previous serological evidence of EBV and had failed standard therapy were given ganciclovir twice daily where it was found that a 2-week course of ganciclovir was effective in some patients [136]. It is to be noted, however, that this study was observational hence the patients were not blinded or randomized. Furthermore, the study sample size was small. 

### 4.7. Phosphodiesterase 5 Inhibitor

Sildenafil is an oral phosphodiesterase-5 (PDE-5) inhibitor and pulmonary vasodilator that is approved for patients with pulmonary arterial hypertension and improves exercise capacity, WHO functional class, and hemodynamics [137]. A study that looked at the effects of sildenafil on exercise capacity in IPF patients with right-sided ventricular dysfunction demonstrated that sildenafil treatment resulted in significantly better exercise capacity and quality of life [138]. On the other hand, a double-blind, randomized, placebo-controlled trial of sildenafil in patients with advance IPF found no benefit of sildenafil on 6-min walk distance [139]. More recently, the STEP-IPF and INSTAGE trials assessed the combination therapy of nintedanib plus sildenafil; both failed to show any benefits of combination therapy over nintedanib alone in the SGRQ and FVC between IPF patients with or without signs of right heart dysfunction at the baseline [140,141]. 

The INCREASE study investigated treprostinil, an inhaled form of PDE-5 inhibitor, in patients with various types of interstitial lung disease (ILD) and associated pulmonary hypertension and found a significant improvement in the mean FVC at 16 weeks compared to the placebo. A sub-analysis conducted on patients with IPF interestingly demonstrated an augmented beneficial effect of inhaled treprostinil compared to the general ILD group [142]. The results are encouraging albeit the short duration of the study, and highlights the potential of inhaled PDE-5 as a therapeutic option for IPF pending further investigation with longer clinical trials. 

## 5. Conclusions

It is without doubt that significant advances have been made from a pharmacological perspective in the treatment of IPF in the last decade with the emergence of the two anti-fibrotic agents pirfenidone and nintedanib, marking a very significant milestone in the battle against this disease. These advances were made possible by the improved understanding of the pathophysiology of the disease. Once thought to be rare and “idiopathic”, the incidence of IPF is increasing globally, most likely due to a refinement in the diagnostic criteria and increased recognition of the disease in clinical settings. Recent acquired knowledge of genetic and non-genetic risk factors contributing to this heterogenous disease have opened avenues for further research to be conducted in developing new drugs or repurposing old drugs to treat this debilitating disease. There are several novel treatments currently in phase 3 clinical trials, whose results are much anticipated by the pulmonary fibrosis community. 

## Data Availability

No new data was created in this article.
